# Comparative Assessment of Blood Lead Levels of Automobile Technicians in Organised and Roadside Garages in Lagos, Nigeria

**DOI:** 10.1155/2015/976563

**Published:** 2015-02-10

**Authors:** Abdulsalam Saliu, Onajole Adebayo, Odeyemi Kofoworola, Ogunowo Babatunde, Abdussalam Ismail

**Affiliations:** ^1^Department of Community Medicine, Ladoke Akintola University of Technology (LAUTECH), Teaching Hospital, Ogbomoso, Oyo State, Nigeria; ^2^Department of Community Health and Primary Care, College of Medicine, University of Lagos, Idi-Araba, Lagos, Nigeria; ^3^Epidemiology Unit, Directorate of Public Health, Lagos State Ministry of Health, Alausa, Ikeja, Lagos, Nigeria

## Abstract

Occupational exposure to lead is common among automobile technicians and constitutes 0.9% of total global health burden with a majority of cases in developing countries. The aim of this study was to determine and compare the blood lead levels of automobile technicians in roadside and organised garages in Lagos State, Nigeria. This was a comparative cross-sectional study. Data were collected using interviewer-administered questionnaires. Physical examinations were conducted and blood was analysed for lead using atomic spectrophotometery. Statistical analyses were performed to compare the median blood lead levels of each group using the independent sample (Mann-Whitney *U*) test. Seventy-three (40.3%) of the organised compared to 59 (34.3%) of the roadside groups had high blood lead levels. The organised group had statistically significant higher median blood lead levels of, 66.0 *µ*g/dL than the roadside 43.5 *µ*g/dL (*P* < 0.05). There was also statistically significant association between high blood lead levels and abnormal discolouration of the mucosa of the mouth in the organised group. Automobile technicians in organised garages in Lagos have higher prevalence of elevated blood lead levels and higher median levels than the roadside group. Preventive strategies against lead exposures should be instituted by the employers and further actions should be taken to minimize exposures, improve work practices, implement engineering controls (e.g., proper ventilation), and ensure the use of personal protective equipment.

## 1. Introduction

The automobile technician workers, among other occupation groups, are exposed to some health hazards. The World Health Organisation (WHO) gave the annual number of non-fatal work-related diseases caused by exposure to hazards and dangerous conditions at the workplace at 160 million of the 2.8 billion global workforces [1, 2]. Lead poisoning is a major potential public health problem throughout the world particularly in developing countries. Intoxication with lead accounted for 0.9% of the total global diseases with between 0.5 and 1.5 million being due to occupational exposure [3].

Lead poisoning or intoxication occurs when people are exposed to lead or chemicals that contain lead. Occupational exposures to lead usually occur through breathing in air that contains lead particles and sometimes by direct ingestion. The common sources of lead exposure also include environmental emissions containing lead [4]. Lead that is emitted into the atmosphere can be inhaled, or be ingested after it settles out of the air [5]. Lead has become widely dispersed throughout the environment because of the number of human uses of it.

Occupational lead poisoning has been a recognised health hazard for more than 2,000 years. Pliny the Elder (as far back as A.D. 23–79) wrote that workers painting ships with native ceruse (white lead) wore loose bags over their faces to avoid breathing of noxious dust [6]. Hippocrates in 370 B.C. attributed a severe case of colic in a worker who extracted metals to lead exposure. The characteristic features of lead toxicity, including anemia, colic, neuropathy, nephropathy, sterility, and coma, were also noted by Hippocrates. The credit for the first undoubted account of clinical manifestations of lead poisoning is to Nikander who in the second century B.C. gave descriptions of pallor, colic, paralysis, and drooping limbs. George Baker established the link between abdominal colic (due to lead poisoning) and the “Devonshire colic” a symptom suffered by people of Devon (an area of Britain at that time) who drank cider made in presses that were lined with lead in the 18th century [7]. Ramazzini described the dangers of poisoning from lead used by potters in their glaze [5]. Hamilton investigated white lead employing careful and extensive use of hospital records to demonstrate the connection between specific illnesses and occupations and through investigation of factories to learn which industrial process used dangerous chemicals [8]. The prevalence of lead poisoning in ancient times has been adequately reported and it has been suggested that Rome actually fell because of the prevalence of lead poisoning in her citizens [9, 10].

As far back as 1800s, there was an increasing recognition of hazards to health associated with lead. Historically, there was an epidemic of lead poisoning in an automobile industry in the United States of America as far back as 1924 [11]. Generally, lead poisoning manifests as elevated blood lead levels and is particularly a problem among the socially and economically deprived persons. The magnitude of the problem is not known especially in developing countries. In adults, occupational exposure is the main cause of lead poisoning [12]. These occupational exposures have adverse effects on their health and that of their families. Families of workers may also be exposed to higher levels of lead when workers bring home lead dust on their work cloths [13].

In Nigeria, factory and small scale industry workers including automobile technicians are often exposed to lead. The automobile technicians exposed to lead include battery manufacturers and repairers, panel beaters, spray painters, and radiator repairers [14, 15]. There have been reported cases of lead poisoning and deaths from ingestion and inhalation of gasoline among mechanics [6, 15–17]. Automobile mechanics were also reported to suck petrol and use it to wash hands and this leads to absorption of tetraethyl lead through mucosa, and this, with inorganic lead from exhaust fumes, may lead to elevated blood levels [6, 18]. In certain areas where the habit of feeding with barehands and poor personal hygiene exists, the contaminated hands and mouths of automobile technicians are also important sources of occupational lead poisoning [19, 20]. It is also known that some of these workers who consume their meals in the workshops are greatly exposed to lead [17, 21]. Exposure among workers can also occur through the habit of chewing of lead containing connective wires [22]. Lead poisoning of high magnitude arises from occupational and environmental exposures probably due to high gasoline lead [23]. Thus, the lead absorbed in the course of occupational exposure is superimposed on lead absorbed from other means [22, 24].

The effects of lead are the same irrespective of the route of entry. Lead intoxication is a slowly progressive condition which is difficult to detect but could manifest with nonspecific symptoms such as irritability, stomach ache, diarrhoea, colic, distractibility, and lethargy [25]. Acute lead poisoning from uncontrolled occupational exposures could result in fatalities while chronic overexposure causes severe damage to the haematopoietic, nervous, cardiovascular, urinary, and reproductive systems [20]. In adults but particularly in children, the main target for lead toxicity is the nervous system where it interferes with its development causing potentially permanent learning and behaviour disorders. The factors that determine the toxicity of lead to an individual include age, sex, dose, duration of exposure, mode of contact, diet, lifestyle, state of health, and other chemicals that one is exposed to [26]. The frequency and severity of symptoms increase with the concentration of lead in the blood.

Many laboratories do not have the capacity for assessing blood lead and this contributes to making the estimates of the health burden of lead poisoning difficult to ascertain. Reports from the United States of America account for the majority of the available data on occupational lead poisoning [27]. In developing countries, there is dearth of information on lead poisoning. In a study in Egypt, lack of data on lead pollution to enable estimation of lead intake in the country was expressed [28]. This is probably true of Nigeria as there are presently no national surveys of blood lead levels in the general population [24]. However, there is overwhelming evidence that lead pollution is on the rise in Nigeria and the exposure routes are as varied as its toxicity [29]. A recent outbreak of occupational lead poisoning related to gold mining led to the death of about 200 children and the affliction of over 18,000 people in seven villages in Zamfara State of Nigeria [12].

Occupational guidelines and regulations worldwide argue for Blood Lead Levels (BLLs) of 40 *μ*g/dL as the absolute highest which should be permitted, with 25 *μ*g/dL (and below) being as preferred target levels for exposed adults. BLLs above 50 *μ*g/dL are astronomical and corrective action must be taken immediately to prevent any additional exposures to workers having such high blood lead concentrations.

Numerous activities involving lead and its products are carried out by the automobile technicians. These activities include spray painting, panel beating, metal cutting, radiator repair, battery charging, and welding as well as repair works by mechanics. Although many African countries have outlawed leaded petrol, there is no assurance that leaded petrol is not still being sold in Nigeria [30]. Leaded petrol and petroleum products may still be utilized by automobile technicians in their occupation. Therefore, exposures to lead in their workplace continue to be a significant public health problem. The findings of few studies in Nigeria showed a strong association between exposure to lead and the prevalence of adverse health effects [23, 31, 32]. Lead intoxication was reported in 40% of lead-acid battery workers in Lagos metropolis as far back as 1971 [30]. Consistent observations have also shown that occupational hazards associated with lead processing including long term inhalation of lead fumes from smelter, emissions, inhalation, or oral ingestion of lead dust and dermal absorption of dissolved organic lead could cause elevated blood lead levels [20, 23, 33]. Personal Protective Equipment (PPE) is scarcely used by automobile mechanics with protective overalls being the commonest used, if at all [33].

Basically, the automobile technicians in Lagos State exist in two distinct groups, either as organised in formal environment, or informal as in roadside garages. For the purpose of this study, the roadside automobile technicians constitute a large group, especially in South West Nigeria, that work in all types of makeshift outdoor locations, usually in undeveloped plots scattered along and close to major roads and streets in the towns and cities [32]. In contrast, the organised group exist in workshops owned by private and corporate organizations and are viewed to be protected by Nigerian labour and factory laws [34]. In view of the increasing number of automobile technicians and multitude of mechanic garages springing up in various towns and cities in Nigeria, this study is of public health importance. This study was carried out to compare the blood lead levels of automobile technicians in organised and roadside garages in Lagos, Nigeria.

In Lagos, there are large mechanic villages and roadside workshops with large pools of automobile technicians of different trades who provide needed services to this densely populated city with a relatively high number of motor vehicles. Anecdotal information showed that it is typical of automobile workshops in Nigeria to find groups of artisans complementing each other's services with preponderance of automobile mechanics in most workshops. Apart from this group on the roadside and mechanic villages, there are other automobile technicians who are those in organised garages.

## 2. Material and Methods

This study was a comparative cross-sectional survey carried out in Island and Mainland Local Government Areas (LGAs) of Lagos State between October and December 2011. Lagos State is located in the South-Western part of Nigeria at latitude 6-degree 27 inches North of the Equator and longitude 3-degree 24 inches E of the Greenwich Meridian. Lagos is the commercial capital of Nigeria where most of the nation's wealth and economic activity are concentrated. The city has one of the largest and most extensive road networks in West Africa. There are about 2,600 Km of roads in Lagos that are frequently congested with over one million vehicles plying them on daily basis [35]. The Urban Lagos has the highest vehicular density of over 222 vehicles/Km against national average of 11 vehicles/Km. Lagos State accounts for over 40% of all new vehicle registration and about 40% of the total fuel consumption in the country.

The roadside automobile technicians in Lagos State constituted themselves into associations, one of which is the Motor Mechanics Automobile Technicians Association of Nigeria (MOMTAN). The association operates as zones with their State secretariat at 12, Cemetry road, Okesuna, Lagos. These zones are further divided into divisions which are further split into branches. Each branch consists of a number of workshops.

The study population included 351 males and two females (353 respondents) who were automobile technicians in Zone 3 (Obalende zone) of MOMTAN, Lagos State branch. A multistage sampling method was carried out to select 172 males from registered members (masters only) of roadside automobile technicians of Zone 3 and 179 males and 2 females from organised garages within the same zone.

The sample size was determined using the formula *n* = (*Z*_*α*_ + *Z*_*β*_)^2^ × 2 × *P* × (100 − *P*)/*d* ^2^ with the level of significance (*α*) and power (1 − *β*) being set at 5% and 80%, respectively [36]. The *n* was the desired sample size, *Z*_*α*_ percentage point of the normal distribution = 1.96, and *Z*_*β*_ one-sided percentage point of the normal distribution corresponding to 100% = 0.04. The *P* = *p*_1_ + *p*_2_ where *p*_1_ was the proportion of roadside lead workers (including automobile technicians) in previous literature who had blood lead level (due to occupational exposure) equal to or above normal blood lead level [37]. The *p*_2_ was the proportion of automobile repair technicians in modern (organised group) automotive refinishing industry in previous literature who had blood lead level (due to occupational exposure) equal to or above normal blood lead level [2]. The study provided for 85% response rate and approximated 170 respondents for each group.

A pretested, interviewer-administered questionnaire was the tool for data collection. The questionnaire was pretested among the roadside automobile technicians in Zone 1 (Ikeja zone) of MOMTAN and those in organised garages within the same Zone and necessary amendments made subsequently. The Zone 1 is similar in sociodemographic characteristics to Zone 3.

Physical examinations were carried out by the researchers and venous blood samples were collected by phlebotomists. Interviewers had a common training programme which included standardization of methods of questionnaire administration, appropriate procedures for collection of blood samples, and disposal of sharps and medical wastes. Emphasis was placed on standard universal precautionary measures so as to avoid accidental injury. A soft tubing tourniquet was tied to the upper arm of the subjects to enable veins to be seen and the puncture sites were cleaned with 70% ethanol and allowed to dry. The subjects were asked to clench their fist. With gloved hands, using the index finger, suitable veins were felt with the thumb of the left hand holding down the skin below the antecubital fossa. Blood samples from each subject were taken using a sterile plastic 5 mL syringe attached to 21 G disposable needle. Five mls of blood samples was collected from each subject into lithium-free heparinized bottles. All the sample bottles were tightly covered and well labelled using the codes on each questionnaire for proper identification to match the subjects prior to storage in GIO-STYLE cold boxes with ice packs. The samples were dispatched for analysis at the end of each day or stored in a refrigerator in the case of those collected over the weekends. All medical wastes such as used swabs and gloves were collected into appropriately labelled disposable bags. Sharp objects were put into disposable biological waste containers (Kojak Safety box) and sealed as per manufacturer's instructions.

All the blood samples collected from subjects from both groups were analysed at the Department of Chemistry of the University of Lagos laboratory by an industrial hygiene-environmental health laboratory scientist accredited by the Nigerian National Council for Industrial Hygiene and Environmental scientist. The blood samples were analysed for lead content by means of a graphite-furnace electrothermal atomic absorption spectrophotomer (PerkinElmer-A-Analyst-200) adjusted to a wavelength of 283.0 nm generated from a coded Lumina single-element Hollow Cathode Lamps (HCLs) and Electrodes-less Discharge Lamps (EDLs). Care was taken to prevent contamination from the environment of blood specimens during sample preparation and analysis. The instrument was adjusted as follows: wavelength of 283.3 nm, band pass of 1.0 mm, reciprocal sensitivity of 0.06 *μ*g/dL, detection limit of 0.05 *μ*g/dL, and optimum working range of 2.5.

A portion (2 mL of each of the blood sample) was transferred using calibrated automatic micropipette into 100 mL volumetric flasks and diluted to 100 mL volume using Aqua Regia (combination of Nitric Acid and HCL in the ratio 1 : 3) solution to digest the samples and the contents of each flask were thoroughly shaken to mix. The lead levels in the diluted solutions were measured by introducing the sample directly into a graphite tube, which is then heated in a programmed series of steps to remove the solvent and major matrix components and to atomize the remaining sample. All of the analyte is atomized, and the atoms are retained within the tube (and the light path, which passes through the tube) for an extended period of time.

High and low controls were run for every batch of samples analyzed which ensured quality assurance and control. Further quality control was achieved with two laboratory scientists simultaneously carrying out the same sample analysis with two different machines at the same time. All procedures carried out on the samples of subjects from organised group were also applied to the roadside group under the same conditions. The performance of the spectrophotometers was rechecked on each batch ran through internal calibration with the standard. Batch of some samples taken at random were sent to National Institute for Medical Research, Yaba, Lagos to further ensure quality control. All results were confirmed to be within the expected control ranges. The cost of the estimation of the blood lead levels was borne by the investigator.

Data entry and analysis were carried out using Statistical Package for Social Sciences (SPSS) Version 17.0. Descriptive statistics were represented by frequency tables, proportions, and percentages. The population was discovered to be skewed with wide ranging of blood lead levels from 0 to 133 *μ*g/dL and large standard deviations which made the mean values inappropriate measure of central tendency. Consequently, the medians and ranges of blood lead levels in both groups were calculated. The median blood lead levels of each group were compared using the two-sided *P* value of independent sample Mann-Whitney* U* Test. Statistical analysis of differences between proportions was carried out using the Pearson Chi square. Statistical analysis was set at *P* < 0.05 for all values of the Chi square and Mann-Whitney* U* Test.

The Ethics Committee of the Lagos University Teaching Hospital, Lagos, approved the study. The aim and objectives of the study were clearly stated in a letter to the MOMTAN executives requesting for permission to conduct the research within the association. Written informed consent duly signed by each participant was obtained before blood samples were taken.

## 3. Results

A total of 353 respondents comprising 172 males from the roadside and 179 males and 2 females from organised group participated in the study. Their mean ages were 44.60 ± 10.52 and 41.80 ± 8.6 years for the roadside and the organised group, respectively. The sociodemographic profile of the subjects is shown in Table 1.

Table 2 shows the respondent's awareness of lead as hazards in their professions. A higher proportion 128 (70.7%) of the organised group claimed statistically significant higher awareness than that of the roadside group, 102 (59.3%).

Blood lead levels in excess of 40 *μ*g/dL are taken as the absolute highest which should be permitted globally. Of the organised group, 108 (59.7%) had less than the absolute values while 73 (40.3%) had high blood lead levels compared to the roadside group with 59 (34.3%) being with above highest value and 113 (65.7%) being with less values, respectively. These findings were not statistically significant, *P* > 0.05 (Table 3).

The median blood lead levels of the organised automobile technicians, 66.0 *μ*g/dL, were higher than those of the roadside group, 43.5 *μ*g/dL. The Mann-Whitney* U* Test revealed significant differences in the blood lead levels of the organised subjects (Median = 66 *μ*g/dL, *n* = 181) and roadside (Median = 43.5, *n* = 181),* U* = 13652.5,* z* = −1.263, *P* = 0.04, and *r* = 0.11. The difference in their median blood lead levels was thus statistically significant, *P* < 0.05. In this study, it means that there was an increased level of exposure of these workers to lead (Table 4).

Among those that had pallor, 24.0% of organised group and 13% of the roadside group had high blood lead levels. Of those that had jaundice, 13.4% of the organised group compared to 21.0% of the roadside group had high blood lead levels. Among those that had abnormal pigmentation of the mucous membrane of the mouth, 25.0% were of the organised group while 15.0% of those of the greater blood lead levels. This last finding was statistically significant, *P* < 0.05. In the organised group, out of the 71 with abnormal discolouration of mucous membrane of the mouth (Burton Line), 40.8% had high blood lead level (Table 5). This finding was statistically significant, *P* < 0.05.

## 4. Discussion

Three hundred and fifty-three respondents with mean age of 44.6 ± 10.5 and 41.8 ± 8.6 years in roadside and organised groups, respectively, and a predominantly male population participated in the study. A wider age range (13–72 years) was reported in roadside automobile mechanics in Ibadan and 18–66 years in Enugu, respectively [27, 32]. This was because apprentices were included in these studies. In another study in two metropolitan cities of South West Nigeria where apprentices were included, an age range of 29.0 ± 0.1 years was reported for battery charging artisans [38]. In Turkey, a lower mean age of 35.0 ± 9.0 years was reported among workers from several workplaces containing lead [39].

A majority of the automobile technicians that participated in this study were males (99.4%) with only 2 females representing 0.6% (Table 1). All the technicians in the roadside and majority 179 (98.9%) in the organised group were males. There were 2 females (1.1%) in the organised group. This is not an unusual finding as the nature of the job is physically exerting and it is socially viewed as a “man's job” but few females are springing up into the profession in the organised set-ups [40]. Most studies in the past had never reported the involvement of females in these trades [3, 31, 41]. However, a study of battery workers in Lagos reported 2.7% of females [42].

There was no statistically significant difference in the proportion of subjects with blood lead level and the marital status of automobile technicians in roadside and organised groups. Single subjects may have been expected to exhibit an irregular lifestyle compared to married ones; they may be expected to behave more carelessly in the utilization of PPE which could lead to higher blood lead levels in them. In the present study, there was no statistically significant difference regarding the prevalence of high blood lead level between single and married workers, *P* > 0.05.

The largest proportion of the respondents in the roadside group (66.3%) had only primary education and 5.2% had none (Table 2). Very few (1.2%) had postsecondary education. However in the organised group majority, 47.0%, completed secondary or technical education and a large proportion, 26.5%, had postsecondary education. The difference in the two groups was statistically significant, *P* < 0.05. The 1.2% recorded for postsecondary education in roadside group agreed with the 1.1% and 1.2% in previous studies in Lagos and Anambra State, respectively [3, 43]. Similar findings in respect of roadside group were also reported from Ibadan, Ilorin, and Tanzania [27, 44, 45]. In Tanzania, a majority of workers in informal sector (including vehicle repair workshops) were primary school leavers [42].

Automobile mechanics formed the bulk, 152 (43%) of automobile technicians seen in both groups combined with 91 (50.3%) in organised group and 61 (35.5%) in roadside group, respectively. Similar findings were reported in Ibadan 68%, Lagos 55%, and Ile-Ife 47% [3, 4, 32]. The differences in the proportion of automobile technicians in both groups were statistically significant, *P* < 0.05.

A majority 260 (73.7%) of these workers had spent over 10 years on the job. A large proportion 147 (85.5%) of the subjects in the roadside and 113 (62.4%) of the organised group had spent more than 10 years on the job as “masters.” Twenty-three (12.7%) of the roadside group compared to 10 (5.8%) of the organised group had spent 2–5 years. This observation was found to be statistically significant, *P* < 0.05. A similar finding in South West Nigeria reported that 53 to 60% had 5 to 10 years of experience on the job and apprentices were also not included [4].

A majority of the workers in the organised group (93.4%) spent between 6 and 8 hours at work in a day and worked 6 to 7 days in a week compared with the roadside group where majority (70%) spent more than 10 hours in a day at work. They resumed work between 7.30 and 8.00 o'clock in the morning and close between 7.00 and 8.00 p.m. These observations were statistically significant, *P* < 0.05. The mean time spent at work was 5.57 ± 0.79 hours for the roadside group while the organised group was 5.43 ± 0.25 hours. In a study in Turkey, the mean time was reported to be 8.88 ± 1.52 hours per day but varied between 7.5 and 12 hours with 44.9% of the workers working more than 8 hours a day [35]. The median and mode were similar but the minimum time spent per day by roadside group was 2 hours while for the organised group it was 4 hours.

Generally, a majority (92%) claimed awareness of hazards. Of the roadside group, 156 (90.7%) had general awareness which is similar to 168 (92.8%) in the organised group. Specifically, 102 (59.3%) of roadside and 128 (70.7%) of the organised groups claimed awareness of lead exposure as being a hazard in their occupation. This finding was statistically significant, *P* < 0.05 (Table 2).

It must also be underscored that the blood lead levels which were found in both worker groups are extremely high. Occupational guidelines and regulations worldwide argue for BLLs of 40 *μ*g/dL as the absolute highest which should be permitted, with 25 *μ*g/dL (and below) being as preferred target levels for exposed adults. Blood lead level above 50 *μ*g/dL is astronomical and corrective action must be taken immediately to prevent any additional exposures to workers having such high blood lead concentrations. The results in the current study showed significantly higher prevalence of high blood lead levels among the organised group compared to the roadside group (Table 3).

Of the organised group, 108 (59.7%) had normal blood lead level while 73 (40.3%) had high blood lead levels compared to the roadside group, with 113 (65.7%) and 59 (34.3%) being with normal and high blood lead levels, respectively. These findings were not statistically significant, *P* > 0.05 (Table 3). A similar percentage (37.5%) distribution as in this study was found among roadside lead automobile workers in Benin [37]. The proportion for the organised group was however higher than that reported for automotive technicians in an organised setting in Rhode Island (22%) [2]. Other studies from Sudan and Eskisehir, Turkey, reported 23% and 10.2%, respectively [39, 46]. Higher values to those recorded in this study were reported in roadside mechanics in South West Nigeria, (75%) and among battery chargers in Lagos (51.6%) [38, 42]. Other previous studies also reported higher prevalence of high blood lead levels in lead workers [45, 47].

Since the blood lead levels in the study populations were skewed with a wide range from zero to 133 *μ*g/dL and with large standard deviation (42.0 and 41.1 *μ*g/dL for organised and roadside groups resp.), the mean values were found to be inappropriate measure of central tendency and the median blood lead level was therefore utilized. The median blood lead level in the organised group was 66.0 (0.0–133.0) *μ*g/dL and 43.5 (0.0–130.0) *μ*g/dL for the roadside group. There were grouping of the median values and comparison of the two groups using Mann-Whitney* U* Test (Table 4). The Mann-Whitney* U* Test revealed statistically significant difference in the blood lead levels of organised and roadside groups, (*P* < 0.05). The values recorded for these subjects were far in excess of the higher limit of 40 *μ*g/dL of blood lead level permitted by WHO for adults in the general population and a normal blood lead of 10–25 *μ*g/dL exists in adults without occupational exposure [24, 48, 49]. In this study, it means that there was an increased level of exposure of these workers to lead. These median values were higher than 14.1 (7.5–56) that was reported in a study in Basra, Iraq [50]. It could be inferred that the lower value obtained in the studies outside Nigeria was due to the fact that some forms of biological monitoring and protective measures were expected to be in place.

Those with very high values were counselled to quit the trade and referred to Department of Chemical Pathology, Lagos University Teaching Hospital for proper management. Also, further actions were taken to minimize exposures, improve work practices, implement engineering controls (e.g., through improve ventilation), and use personal protective equipment.

The higher median blood lead level in the organised group as compared to roadside group was rather surprising but it could be that higher lead exposure rates occurred in the organised group due to the warehouse-like workshops which were enclosed in most cases. It was also observed that there was an ineffective local ventilation control system in most of the workshops in organised garages. The majority of the roadside workers worked in open spaces which could offer greater dilution to the atmospheric lead. Another reason could be due to inefficient administrative and engineering control measures and lack of proper utilization of PPE. Previous studies reported low use of PPE by automobile technician workers [3, 32, 42]. Further studies to identify factors for the observed difference are strongly advocated.

The comparison of associations between prevalence of high blood lead level and age groups of respondents showed significant association with subjects within age group 20–29 years and high blood lead levels, *P* < 0.05. A study conducted in Turkey revealed a similar result [50]. However, a study performed between 1990 and 2000 on labourers working in places where lead containing materials were used showed an increase of lead poisoning risk with advanced age [51].

The prevalence of high blood lead level was determined to be higher in roadside technicians who had spent over than 10 years on the job (36.1%) while the lowest level (20.0%) was found in those that had spent 2–5 years. In the organised group, similar findings were recorded with the highest being among those that had spent over than 10 years (44.0%) but the lowest was among those that had spent 5–10 years. Previous studies abroad showed prevalence of high blood lead level in workers who worked less than 1 year [52, 53]. In this study, duration at work was not found to be associated with high blood lead level, *P* > 0.05.

In the organised group, out of the 71 with abnormal discolouration of mucous membrane of the mouth (Burton Line), 40.8% had high blood lead level (Table 5). This finding was statistically significant, *P* < 0.05. A study conducted in Sudan revealed Burton lines in 2% of lead workers [46]. In Benin, none of the subject had Burton line [37]. It was observed that this line commonly occurred on diseased gums and its presence may not necessarily indicate that overexposure had occurred. Burton lines have been formed after exposure to cadmium, bismuth, copper, mercury, zinc, and lead [51, 54].

## 5. Conclusion and Recommendations

The results from this study revealed a high prevalence of elevated blood lead levels among organised and roadside automobile technicians. Overall, our findings revealed a high level of prevalence of blood lead in automobile technicians in Lagos State with higher level in the organised sector. The median blood lead level of the organised group was found to be statistically significantly higher than that of roadside group, *P* < 0.05. This study has provided an insight into the effects of lead exposure to this group of professionals in Lagos State, Nigeria. The blood lead levels of some subjects were found to be far above the maximum permissible level and it is therefore safe to conclude that lead toxicity is a problem that does occur among automobile technicians in Nigeria. The study also demonstrates that the blood lead levels of automobile technicians are positively influenced by their occupational practices and workers are therefore in danger of exposure to toxicity. The study further showed a higher blood lead level in organised group that were supposedly regulated by the Occupational Safety and Health Act of the Federal Republic of Nigeria as opposed to the roadside group in the informal sector. There is the need to enforce the Factories Act and carry out regular supervisory checks to ensure adherence to the provisions of labour laws. The Act may actually not meet the real needs of the workers and therefore should be amended to meet contemporary challenges. Furthermore, it is also important to recognize that, in addition to research, good health promotion and protection measures should be given to the automobile technicians in Lagos State. There is also the need for concerted effort on the part of health workers to intensify campaign on the ban importation of leaded petrol into the country so as to reduce the amount of organic lead exposure. Finally, actions should be directed at minimizing exposures, improving work practices, implementing engineering controls (e.g., proper ventilation), encouraging the use of personal protective equipments.

## Figures and Tables

**Table 1:** Sociodemographic characteristics of respondents.

Variables	Roadside (*n* = 172)	Organised (*n* = 181)		Statistical analysis	
	Freq. (%)	Freq. (%)	*N* = 353	*X* ^2^	*P* value
Age group (yrs)					
20–29	9 (5.2)	12 (6.6)	21	8.4	0.14
30–39	51 (29.7)	62 (34.3)	113		
40–49	63 (36.6)	71 (39.2)	134		
50–59	33 (19.2)	31 (17.1)	64		
60–69	13 (7.6)	5 (2.8)	18		
70–79	3 (1.7)	0 (0.0)	3		
Sex					
Male	172 (100)	179 (98.9)	351	1.9	0.50
Female	0 (0.0)	2 (1.1)	2		
Marital status					
Single	7 (4.1)	26 (14.4)	33	17.2^**^	0.00^*^
Married	159 (92.4)	155 (85.6)	314		
Divorced	6 (3.4)	0 (0.0)	6		
Education levels					
None	9 (5.2)	2 (1.1)	11	6.2^**^	0.00^*^
Primary/before secondary	115 (66.9)	4 (25.4161)			
Completed secondary/technical	46 (26.7)	85 (47.0)	131		
After sec.	2 (1.2)	48 (26.5)	50		
Subspecialty					
Mechanics	61 (35.5)	91 (25.1)	152	31.9	0.00^*^
Spray painter	43 (25.0)	24 (13.3)	67		
Panel beater	34 (19.8)	20 (11.1)	54		
Upholstery	14 (8.1)	5 (2.8)	19		
Auto electrician	12 (7.0)	22 (12.2)	34		
Battery charger	0 (0.0)	3 (1.7)	3		
Radiator repairer	2 (1.2)	0 (0.0)	2		
Welder	5 (2.9)	15 (8.3)	20		
Others	1 (0.6)	1 (0.6)	2		

^**^Fisher's exact; ^*^ *P* < 0.00.

**Table 2:** Awareness of lead hazards among respondents.

Awareness	Roadside	Organised		Statistical analysis	
	Freq. (%)	Freq. (%)	*n*	*X* ^2^	*P* value
Yes	102 (59.3)	128 (70.7)	230	5.1	0.02^*^
No	70 (40.7)	53 (29.3)	123		

Total	172	181			

^*^ *P* < 0.05.

**Table 3:** Distribution of blood lead levels amongst subjects.

Blood lead levels	Roadside	Organised	*N*	*X* ^2^	*P* value
(*μ*g/dL)					
Preferred				4.9	0.18
0 to <25	88	74	162
Acceptable			
10 to <25	10	24	59
Permissible			
25 to ≤40	15	10	25
Absolute highest			
40 to 49	11	14	25
Astronomical			
≥50	48	59	107

Total	172	181	353		

**Table 4:** Median blood lead levels (*μ*g/dL) of subjects.

Median (range)	Roadside (*n* = 172)	Organised (*n* = 181)	Mann-Whitney *U* Test
	Median (range)	*z* value	*P* value
43.5 (0.0–130.0) *μ*g/dL	66.0 (0.0–133.0) *μ*g/dL	−1.263	0.04^*^

**Table 5:** Blood lead levels by observed clinical features in subjects.

Variables	*n* = 108 (%)	*n* = 73 (%)	*P*	*n* = 113 (%)	*n* = 59 (%)	*P* value
	Normal	High		Normal	High	
Pallor						
Present	47 (69.3)	33 (30.7)	0.82	40 (71.5)	18 (28.5)	0.52
Absent	61 (62.5)	40 (37.5)		73 (52.4)	41 (47.6)	
Jaundice						
Yes	18 (66.6)	9 (33.3)	0.42	26 (65.0)	14 (35.0)	0.92
None	90 (58.4)	64 (41.6)		87 (66.0)	45 (34.0)	
Discolouration of mucous membrane						
Present	42 (57.6)	18 (42.4)	0.04^*^	45 (67.0)	26 (33.0)	0.60
Absent	66 (92.9)	55 (7.1)		68 (56.7)	33 (43.3)	

^*^ *P* < 0.05.
